# Molecular Interaction Mechanism between 2-Mercaptobenzimidazole and Copper-Zinc Superoxide Dismutase

**DOI:** 10.1371/journal.pone.0106003

**Published:** 2014-08-26

**Authors:** Yue Teng, Luyi Zou, Ming Huang, Yadong Chen

**Affiliations:** 1 School of Environmental and Civil Engineering, Jiangnan University, Wuxi, Jiangsu Province, PR China; 2 Laboratory of Molecular Design and Drug Discovery, School of Basic Science, China Pharmaceutical University, Nanjing, Jiangsu Province, PR China; Wake Forest University, United States of America

## Abstract

2-Mercaptobenzimidazole (MBI) is widely utilized as a corrosion inhibitor, copper-plating brightener and rubber accelerator. The residue of MBI in the environment is potentially harmful. In the present work, the toxic interaction of MBI with the important antioxidant enzyme copper-zinc superoxide dismutase (Cu/ZnSOD) was investigated using spectroscopic and molecular docking methods. MBI can interact with Cu/ZnSOD to form an MBI-Cu/ZnSOD complex. The binding constant, number of binding sites and thermodynamic parameters were measured, which indicated that MBI could spontaneously bind with Cu/ZnSOD with one binding site through hydrogen bonds and van der Waals forces. MBI bound into the Cu/ZnSOD interface of two subdomains, which caused some microenvironmental and secondary structure changes of Cu/ZnSOD and further resulted in the inhibition of Cu/ZnSOD activity. This work provides direct evidence at a molecular level to show that exposure to MBI could induce changes in the structure and function of the enzyme Cu/ZnSOD. The estimated methods in this work may be applied to probe molecular interactions of biomacromolecules and other pollutants and drugs.

## Introduction

Aerobic metabolism would generate large quantities of reactive oxygen species (ROS) including superoxide radicals (O_2_^·–^), hydrogen peroxide (H_2_O_2_) and hydroxyl radicals (OH^·^), which readily react with various cellular components and cause widespread damage [1]–[4]. ROS has been identified as an important factor in cancers [5], [6], diabetes [7], aging [8], inflammation [9], arteriosclerosis [10] and sickle cell disease [11]. As the first line of antioxidant systems, superoxide dismutases (SODs), including copper-zinc superoxide dismutase (Cu/ZnSOD), manganese superoxide dismutase (MnSOD) and extracellular superoxide dismutase (ECSOD), play an essential role in the detoxification of ROS [12]. They can catalyze the dismutation of two O_2_^·–^ anions to H_2_O_2_ and molecular oxygen [13]. Among the three families of SODs, Cu/ZnSOD is most commonly used by eukaryotes. The cytosols of virtually all eukaryotic cells contain the enzyme Cu/ZnSOD, which exists as a dimer [14]. When residues of contaminants in the environment enter an organism, they may interact with Cu/ZnSOD and affect the catalytic activity of Cu/ZnSOD in its tissues.

2-Mercaptobenzimidazole (MBI) is an important member of the thioureylene compound family that is applied in various industrial processes such as corrosion inhibition [15], [16], copper-plating brightening [17], rubber acceleration and/or antioxidation [18]. Although the usability of MBI is indisputable, it is known as a toxic and poorly biodegradable pollutant [19]. Therefore, wide use of MBI results in an increase in the probability of its exposure to organisms. Previous studies reported that MBI could be found as a contamination source in rubber plant waste water [20], rivers [21], street runoff [22] and some drugs (the latter can become contaminated from the MBI in the rubber plunger-seals of syringes and/or drug packing containers) [23].

The toxic effects of MBI on experimental animals have been reported. MBI had potent antithyroid toxicity in rats during a 28-day repeated oral dosing [24]. An inhalation toxicity of MBI on rats showed exposure to MBI caused increased thyroid weight, thyroid follicular cell hyperplasia, reduced triiodothyronine and thyroxine levels [25]. It was reported that thioureylene antithyroid compounds blocked the biosynthesis of thyroxine (T4) by inhibiting thyroid peroxidase (TPX) [26]. Yamano et al. investigated the adverse effects of MBI on pregnant rats and their fetuses and observed major fetal malformations. They concluded that maternal toxicity preceded fetal toxicity [27]. However, little work has been conducted that focus on the molecular interactions governing the effect of MBI on antioxidant enzymes. Thus, the purpose of this study was to understand the interaction mechanism of MBI with Cu/ZnSOD by integrating the binding parameters (association and binding forces) of the interaction and the effect of MBI on the conformation of Cu/ZnSOD by using multiple spectroscopic techniques and molecular modeling. The effects of MBI on the activity of Cu/ZnSOD were also investigated. This work provides basic data for clarifying the binding mechanisms of MBI with the enzyme Cu/ZnSOD and is helpful for understanding human health risk of MBI in vivo.

## Materials and Methods

### Reagents

Cu/ZnSOD from porcine erythrocytes was purchased from Biodee Biotechnology Co., Ltd. 2-Mercaptobenzimidazole (MBI), nitroblue tetrazolium (NBT), methionine, riboflavin and EDTA were obtained from Sinopharm Chemical Reagent Co., Ltd. A 0.2 molL^−1^ mixture of phosphate buffer (mixture of NaH_2_PO_4_·2H_2_O and Na_2_HPO_4_·12H_2_O, pH = 7.4) was used to control the pH. All other reagents were of analytical grade and purchased from standard reagent suppliers. Ultrapure water (18.25 MΩ) was used throughout the experiments.

### Apparatus and measurements

#### Fluorescence measurements

All fluorescence spectra were recorded on an RF-5301PC fluorescence spectrophotometer (Shimadzu Japan) with a 1 cm cell. The excitation wavelength was 280 nm. The excitation and emission slit widths were set at 5 nm.

Synchronous fluorescence spectra of Cu/ZnSOD in the absence and presence of MBI were measured (Δ*λ* = 15 nm, *λ*em = 280–330 nm and Δ*λ* = 60 nm, *λ*em = 310–380 nm, respectively) by an RF-5301PC fluorescence spectrophotometer (Shimadzu Japan). The excitation and emission slit widths were set at 5 nm.

#### UV-visible absorption measurements

The absorption spectra were recorded on a double beam UV-6100 spectrophotometer (Mapada, China) equipped with 1.0 cm quartz cells. Slit width was set at 2.0 nm. The wavelength range was 200–260 nm.

#### Circular dichroism (CD) measurements

CD spectra were made on a MOS-450/AF-CD Spectropolarimeter (Bio-Logic, France) in a 1.0-cm cell at room temperature. Bandwidth was 4 nm and scanning speed was 1 nm/2 s.

#### Molecule docking investigation

Docking calculations were carried out using AutoDock 4.2 (developed by The Scripps Research Institute, USA) [28]. Molecular Operating Environment (MOE) version 2007.0902 (developed by Chemical Computing Group Inc, Canada) was used to prepare the structure of MBI and obtain the energy minimized conformation of MBI [29]. As the crystal structure of Cu/ZnSOD from porcine erythrocytes is unavailable in Protein Data Bank and Cu/ZnSOD from porcine erythrocytes has 90.26% homological identity with human Cu/ZnSOD, the crystal structure of human Cu/ZnSOD (1HL4.pdb) was downloaded. A homodimer of the crystal structure was used for docking calculations. With the aid of AutoDock, the ligand root of MBI was detected and rotatable bonds were defined. All hydrogen atoms were added into the Cu/ZnSOD protein model. To recognize the binding sites in Cu/ZnSOD, blind docking was carried out and grid maps of 126×126×126 Å grid points and 0.375 Å spacing were generated. Docking simulations were performed using the Lamarckian genetic algorithm (LGA) search method. Each run of the docking experiment was set to terminate after a maximum of 250,000 energy evaluations and the population size was set to 150. The conformation with the lowest binding free energy was used for further analysis.

#### Cu/ZnSOD enzyme inhibition assay

MBI was dissolved in ultrapure water to form a 1.0×10^−5^ molL^−1^ stock solution. Different volumes (0, 30, 100 and 300 µL) of MBI solutions were taken in vials and each was mixed with 30 µL of 1 µmolL^−1^ Cu/ZnSOD solution and made up to 1 mL using ultrapure water. Then, the enzymes were incubated in MBI for 60, 120 or 240 min at 4°C. The activity of Cu/ZnSOD was measured by monitoring its ability to inhibit the photoreduction of nitroblue tetrazolium (NBT) [30]. Each 3 mL reaction mixture contained 20 mmolL^−1^ phosphate buffer (pH 7.8), 10 µmolL^−1^ EDTA, 13 mmolL^−1^ methionine, 75 mmolL^−1^ NBT, 2 µmolL^−1^ riboflavin and 300 µL of the enzyme mixture with different MBI concentrations. The final concentrations of MBI in the 3 mL reaction mixtures were 0, 30, 100 and 300 nmolL^−1^. Recording the increase in absorbance at 560 nm followed the production of blue formazan at room temperature [31], [32]. One unit of Cu/ZnSOD represents the amount inhibiting the photoreduction of NBT by 50%.

## Results and Discussion

### Characterization of the binding interaction of MBI with Cu/ZnSOD by fluorescence measurements

#### Fluorescence quenching

Because changes of emission spectra can provide information about the structure and dynamics of macromolecules, fluorescence has been widely used to investigate the interaction between proteins and ligands. We utilized fluorescence quenching to study the interaction between Cu/ZnSOD and MBI.

**Figure 1:**
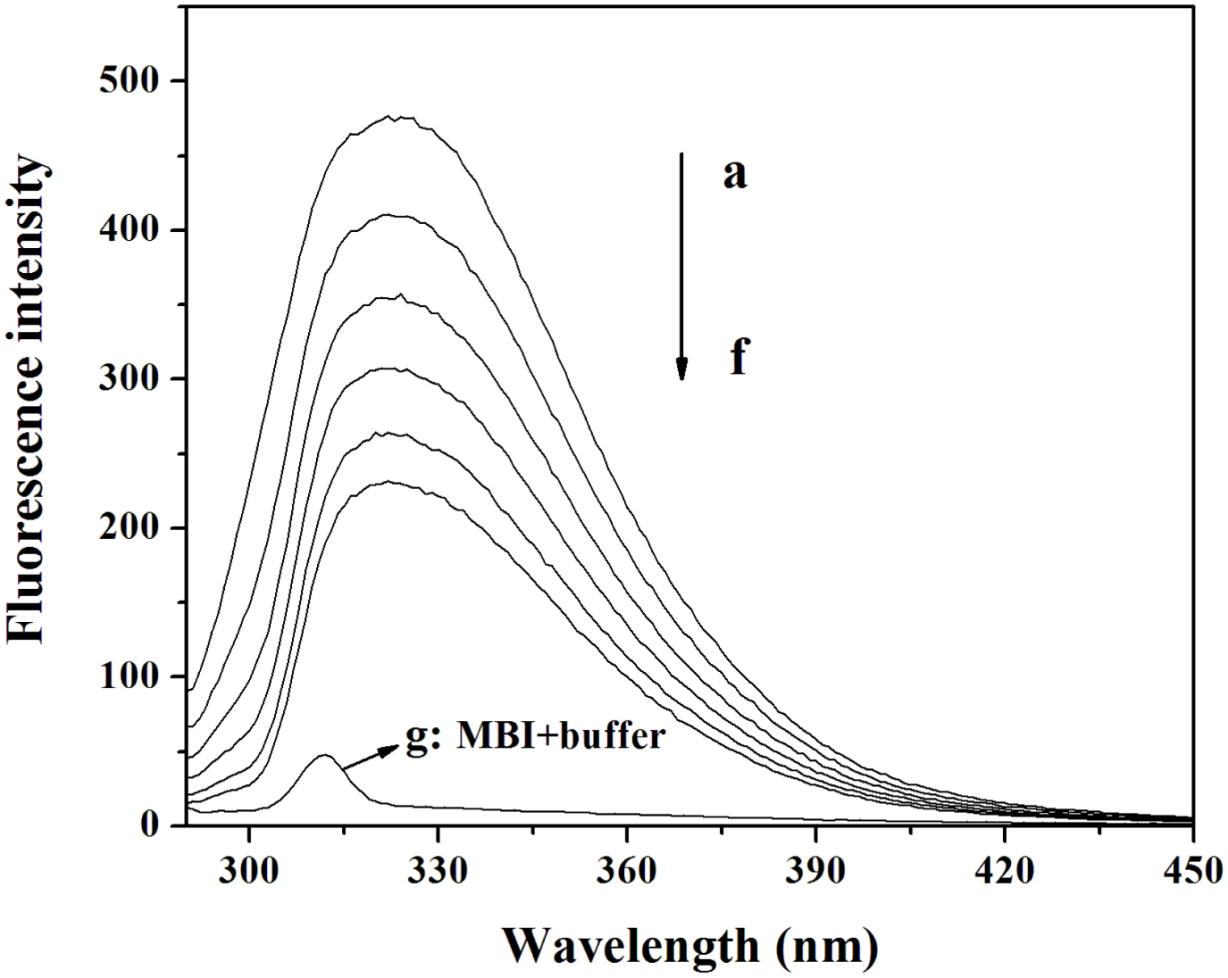
Effect of MBI on Cu/ZnSOD fluorescence. Conditions: Cu/ZnSOD: 3.0×10^−6^ molL^−1^; MBI/(×10^−5^ molL^−1^): (a) 0, (b) 1, (c) 2, (d) 3, (e) 4, (f) 5; (g): MBI (1×10^−5^ molL^−1^) + buffer (0.02 molL^−1^); pH 7.4; *T* = 293 K.

The fluorescence spectra of Cu/ZnSOD at various concentrations of MBI are shown in Figure 1. The fluorescence intensity of Cu/ZnSOD decreased regularly with increasing MBI concentration. The fluorescence of Cu/ZnSOD can be quenched by MBI. It’s reported that Cu/ZnSOD can emit intrinsic fluorescence mainly due to tryptophan and tyrosine residues, and fluorescence quenching was related to structural changes of proteins [30], [33]. So, the fluorescence quenching of Cu/ZnSOD by MBI indicated that MBI may bind to and alter the structure of Cu/ZnSOD.

Quenching mechanisms include static and dynamic quenching. Because higher temperature results in larger diffusion coefficients, the dynamic quenching constants will increase with increasing temperature. In contrast, increased temperature is likely to result in decreased stability of complexes, and thus the static quenching constants are expected to decrease with increasing temperature [34]. To confirm the mechanism, the fluorescence quenching data were analyzed according to the Stern-Volmer equation [35]:
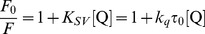
(1)where *F*_0_ and *F* are the fluorescence intensities in the absence and presence of the quencher, respectively. *K*_SV_ is the Stern-Volmer quenching constant, [*Q*] is the concentration of the quencher, *k*_q_ is the quenching rate constant of the biological macromolecule, and *τ*_0_ is the fluorescence lifetime in the absence of quencher.

**Figure 2:**
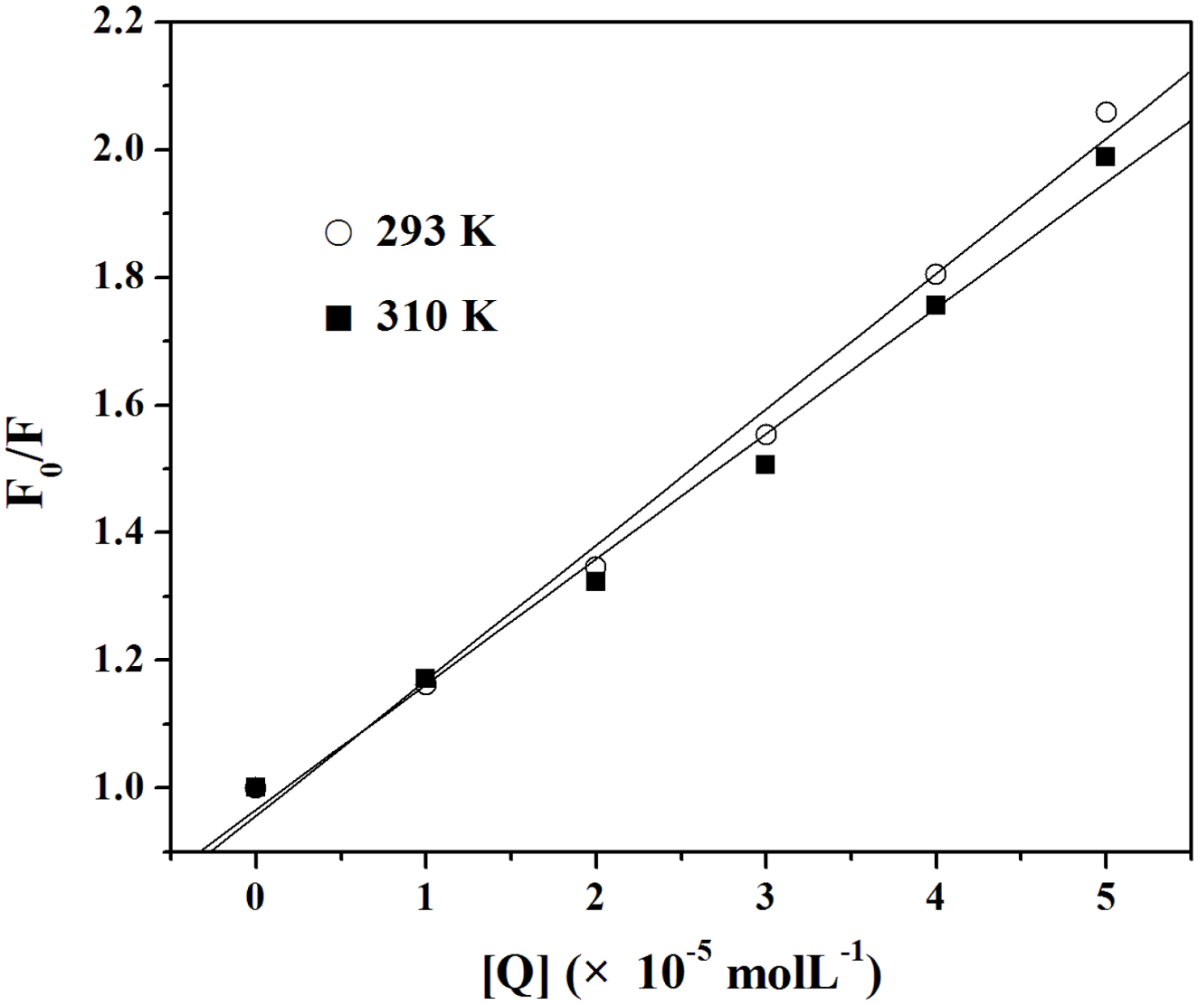
Stern-Volmer plots for the quenching of Cu/ZnSOD by MBI at 293 and 310 K.

Fluorescence data were analyzed according to *F*_0_/*F* versus [*Q*] at 293 and 310 K (Figure 2). Eq. (1) was applied to determine *K*_SV_ (Table 1) by a linear regression plot of *F*_0_/*F* against [*Q*]. The value of *k*_q_ was also obtained (the fluorescence lifetime of the biopolymer (*τ*_0_) is 10^−8 ^s [36]). It can be seen from Table 2 that the *K*_SV_ values decreased with increasing temperature. Moreover, the maximum dynamic quenching constant *k*_q_ of various quenchers is 2.0×10^10^ Lmol^−1^s^−1^ [37]. However, the values of *k*_q_ at 293 and 310 K are greater than 2.0×10^10^ Lmol^−1^s^−1^. The results indicated that the overall quenching was dominated by a static quenching mechanism, and in the process a MBI-Cu/ZnSOD complex was formed.

**Table 1:** Stern-Volmer quenching constants for the interaction of MBI with Cu/ZnSOD at 293 K and 310 K.

*T* (K)	*K*_SV_ (×10^4^ Lmol^−1^)	*k*_q_ (×10^12 ^L mol^−1^s^−1^)	*R* a	*S.D.* b
293	2.12	2.12	0.9960	0.0399
310	1.97	1.97	0.9951	0.0409

a *R* is the correlation coefficient.

b *S.D.* is the standard deviation for the *K*_SV_ values.

**Table 2:** Binding constants and relative thermodynamic parameters of the MBI-Cu/ZnSOD system.

*T* (K)	*K*_a_ (×10^4^ Lmol^−1^)	*n*	*R* a	Δ*H*° (kJmol^−1^)	Δ*S*° (Jmol^−1^K^−1^)	Δ*G*° (kJmol^−1^)
293	10.99	1.17	0.9994	–34.9	–22.5	–28.3
310	5.01	1.10	0.9964		–22.6	–27.9

a *R* is the correlation coefficient for the *K*_a_ values.

#### Binding parameters

For the static quenching interaction, when small ligands bind independently to a set of equivalent sites on a macromolecule, the number of binding sites (*n*) and the binding constant (*K*_a_) can be obtained from the following formula [38]:
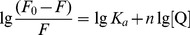
(2)where *F*_0_, *F* and [*Q*] are the same as in Eq. (1), *K*_a_ is the binding constant and *n* is the number of binding sites. *K*_a_ and *n* can be calculated by plotting log[(*F*_0_–*F*)/*F*] versus log[MBI] as shown in Figure S1. The values of *n* and *K*_a_ were calculated and shown in Table 2. The number of binding sites *n* is approximately equal to 1, indicating that there was one binding site in Cu/ZnSOD for MBI during their interaction. Although the values of *K*_a_ decreased with an increase temperature, the value of *K*_a_ in 310 K (310 K is very close to body temperature) was of the order of 10^4^, indicating that a strong interaction existed between MBI and Cu/ZnSOD. Even if a low concentration of MBI is present in organs, MBI can easily interact with Cu/ZnSOD.

#### Determination of the binding forces

The acting forces between small molecular ligands and biomoleculers may involve hydrophobic forces, hydrogen bonds, van der Waals’ interactions and electrostatic forces. When the temperature range is small, the enthalpy change (Δ*H*°) can be considered as a constant and can be approximated from Eq. (3). The free-energy change (Δ*G*°) and the entropy change (Δ*S*°) for the interaction were calculated based on Eq. (4) and (5):
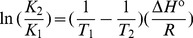
(3)

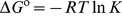
(4)

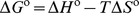
(5)where *K*_1_ and *K*_2_ are the binding constants (analogous to *K*_a_ in Eq. (2)) at *T*_1_ and *T*_2_, and *R* is the universal gas constant. Ross and Subramanian [39] summed up the thermodynamic laws for estimating the type of binding force. That is, if Δ*H*°<0 and Δ*S*°<0, van der Waals and hydrogen bond interactions play the main roles in the binding reaction; if Δ*H*°>0 and Δ*S*°>0, hydrophobic interactions are dominant; if Δ*H*°<0 and Δ*S*°>0, the main forces are electrostatic effects. The thermodynamic parameters of the MBI-Cu/ZnSOD system were listed in Table 3. The Δ*G*° at 293 and 310 K are negative, indicating that the interaction process is spontaneous. Based on the above summary of Ross and Subramanian, the negative Δ*H*° and Δ*S*° mean that hydrogen bonds and van der Waals forces played major roles in the formation of the MBI-Cu/ZnSOD complex.

**Table 3:** Distance between MBI and the neighboring residues (6 Å involved).

MBI atom (atom ID)	Protein atom		Distance (Å)
	Chain	Atom type	
H 12	A	Gly 51 O	1.92
S 11		Asp 52 HN	2.54
H 10		Asn 53 CB	2.65
C 2		Cys 146 O	5.66
C 2		Gly 147 CA	4.74
C 2		Val 148 CG2	3.56
S 11	B	Val 5 CG1	3.34
S 11		Cys 6 CA	3.64
N 7		Val 7 HN	1.77
C 5		Gly 147 CA	3.17
C 4		Val 148 CA	4.96
H 12		Gly 150 HN	3.83

### Identification of the specific binding sites on Cu/ZnSOD

**Figure 3:**
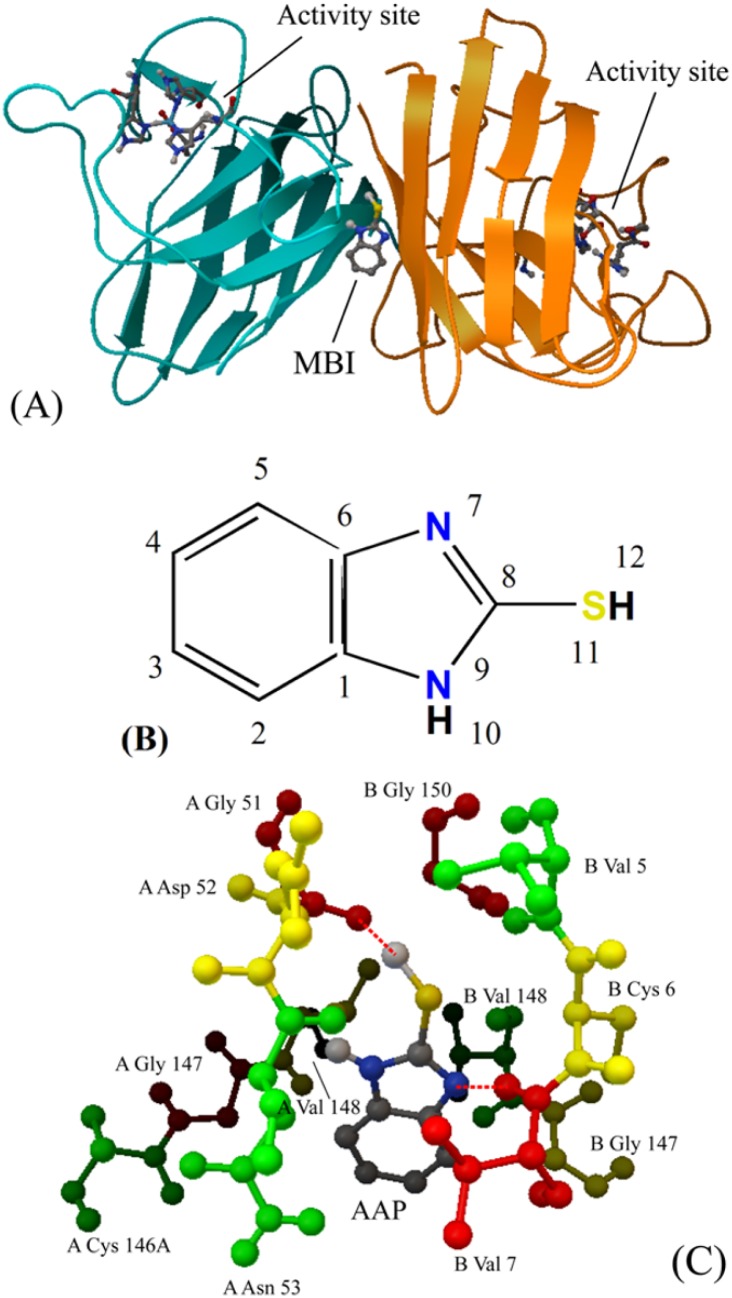
Docking results of the MBI and Cu/ZnSOD system. (A) Binding site of MBI to Cu/ZnSOD. Two subdomains of Cu/ZnSOD are in different colors. (B) 2D structure of MBI with atom numbers. (C) Detailed illustration of the binding between MBI and Cu/ZnSOD. Hydrogen bonds are depicted as red dashed lines. (For interpretation of the references to color in this figure legend, the reader is referred to the web version of the article).

We employed the molecular docking method to identify the binding site of MBI. The exact binding site of MBI on Cu/ZnSOD with the lowest binding free energy is shown in Figure 3A. MBI bound into the interface of the two subdomains of Cu/ZnSOD. The detailed docking results are presented in Figure 3C, and the distances are listed in Table 3. The amino acid residues in the vicinity of this binding site were Gly 51, Asp 52, Asn 53, Cys 146, Gly 147 and Val 148 of chain A and Val 5, Cys 6, Val 7, Gly 147, Val 148 and Gly 150 of chain B. The essential driving forces of the MBI binding of this site were hydrogen bonds and van der Waals forces, and this result was in accordance with that discussed above. As shown in Figure 3C, there is a hydrogen bond between the H atom at position 12 of MBI and the O atom on Gly 51 of chain A. A hydrogen bond also exists between the N atom at position 7 of MBI and the hydrogen atom HN on Val 7 of chain B. Electrostatic interactions and hydrophobic forces also existed, but hydrogen bonds and van der Waal forces played a major role in the binding of MBI to Cu/ZnSOD.

### Investigation on Cu/ZnSOD conformation changes

#### UV-vis absorption spectroscopy

**Figure 4:**
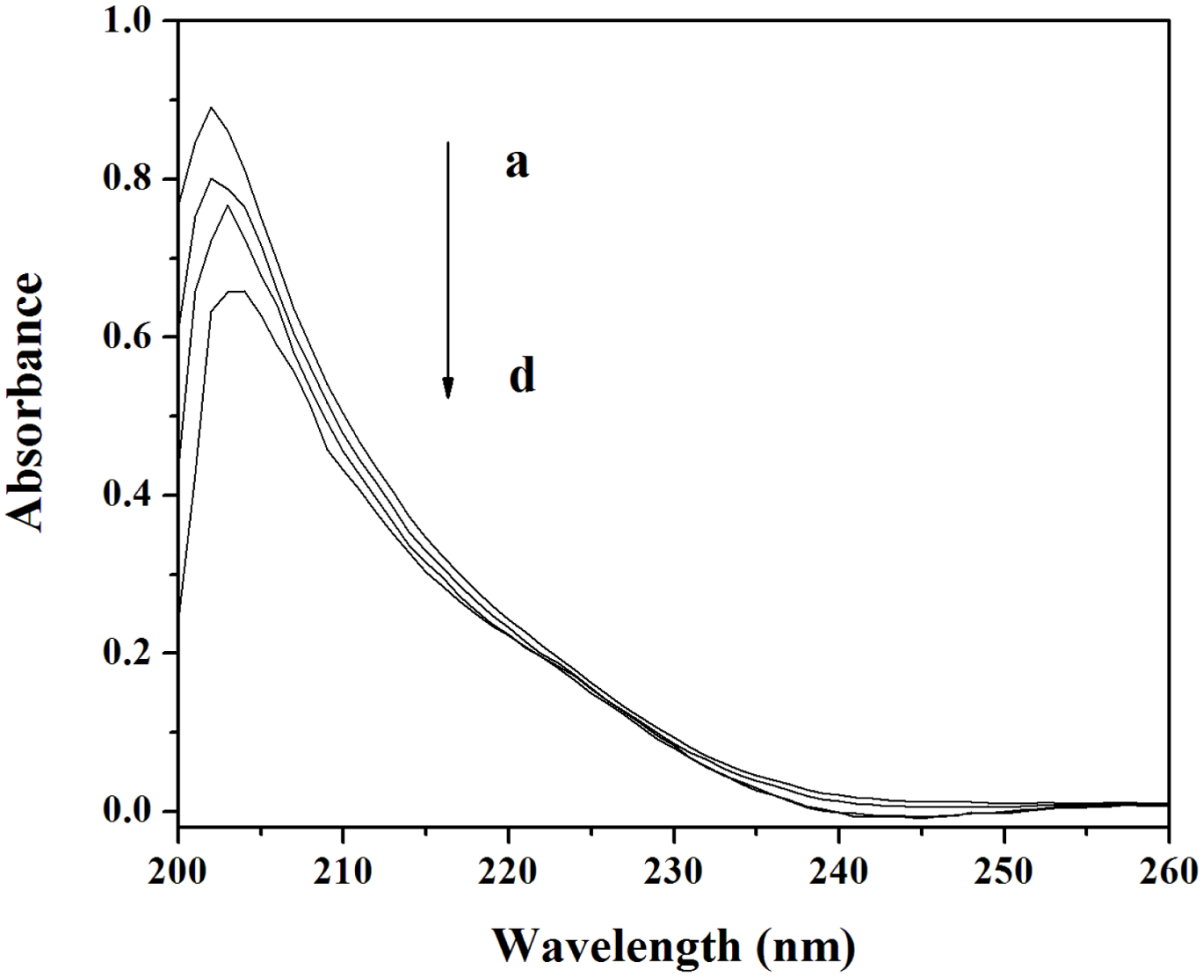
UV-vis spectra of Cu/ZnSOD in the presence of different concentrations of MBI (vs the same concentration of MBI solution). Conditions: Cu/ZnSOD: 1.0×10^−6^ molL^−1^; MBI/(×10^−5^ molL^−1^): (a) 0, (b) 1, (c) 2, (d) 3; pH 7.4; *T* = 293 K.

UV-visible absorption spectroscopy can be used to explore protein structural changes and to investigate protein-ligand complex formation. The UV-vis absorption spectra of Cu/ZnSOD with various amounts of MBI are shown in Figure 4. The strong absorption peak at approximately 202 nm reflects the framework conformation of the protein [30]. When the concentraion of MBI increased, the absorbance of Cu/ZnSOD decreased, and the maximum peak position of MBI-Cu/ZnSOD was red-shifted by 2 nm. The results indicated that the interaction between MBI and Cu/ZnSOD led to the loosening and unfolding of the protein skeleton [40].

#### Synchronous fluorescence

Synchronous fluorescence spectroscopy can give information about the molecular microenvironment in the vicinity of chromophores such as tryptophan and tyrosine because the shifts in the emission maximum are related to the changes in the polarity of their environment. When the wavelength interval (Δ*λ*) is fixed at 15 or 60 nm, the synchronous fluorescence gives characteristic information of tyrosine residues or tryptophan residues, respectively [41].

**Figure 5:**
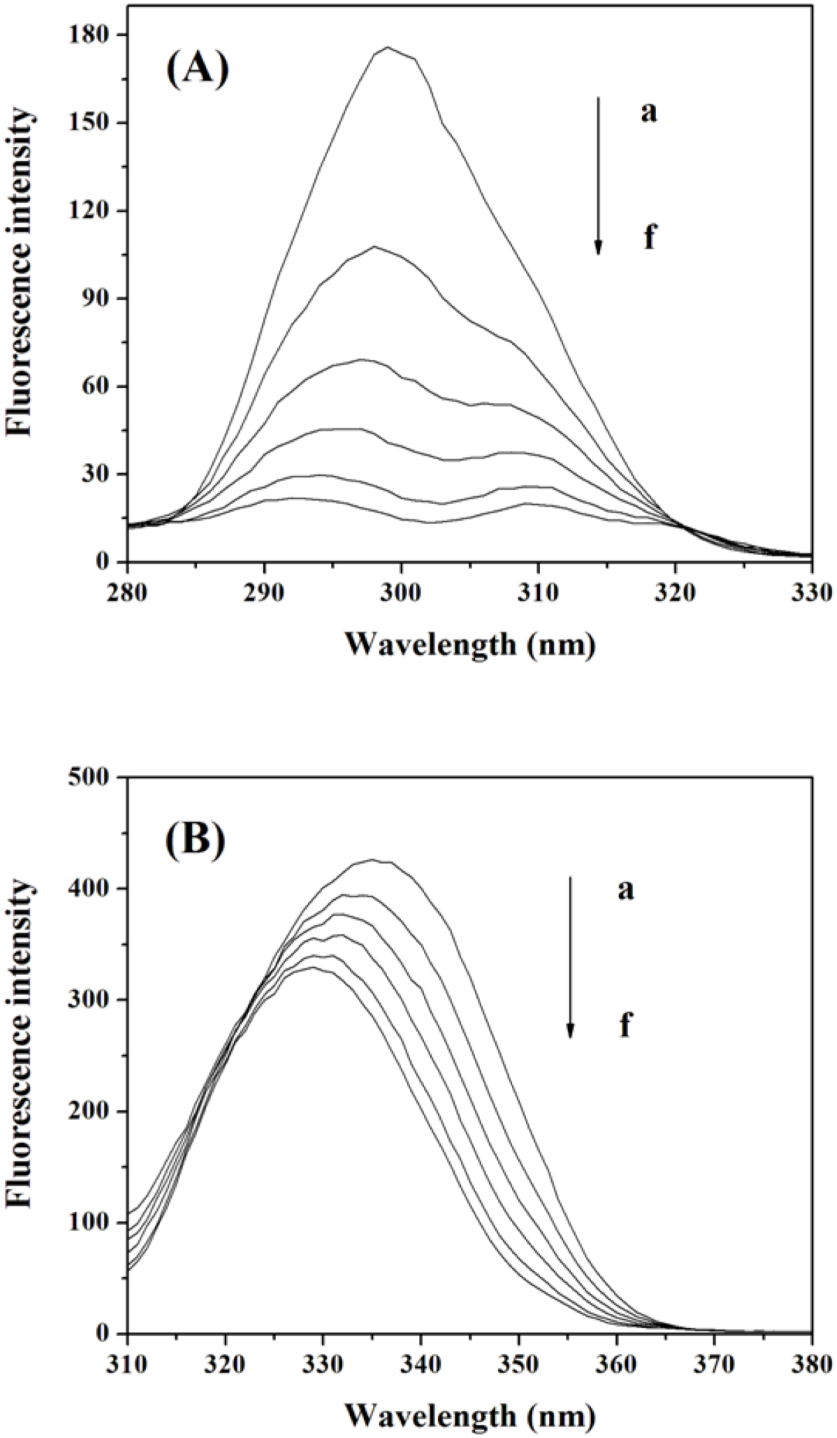
Synchronous fluorescence spectra of Cu/ZnSOD (A) Δ*λ* = 15 nm and (B) Δ*λ* = 60 nm. Conditions: Cu/ZnSOD: 3.0×10^−6^ molL^−1^; MBI/(×10^−5^ molL^−1^): (a) 0, (b) 1, (c) 2, (d) 3, (e) 4, (f) 5; pH 7.4; *T* = 293 K.

**Figure 6:**
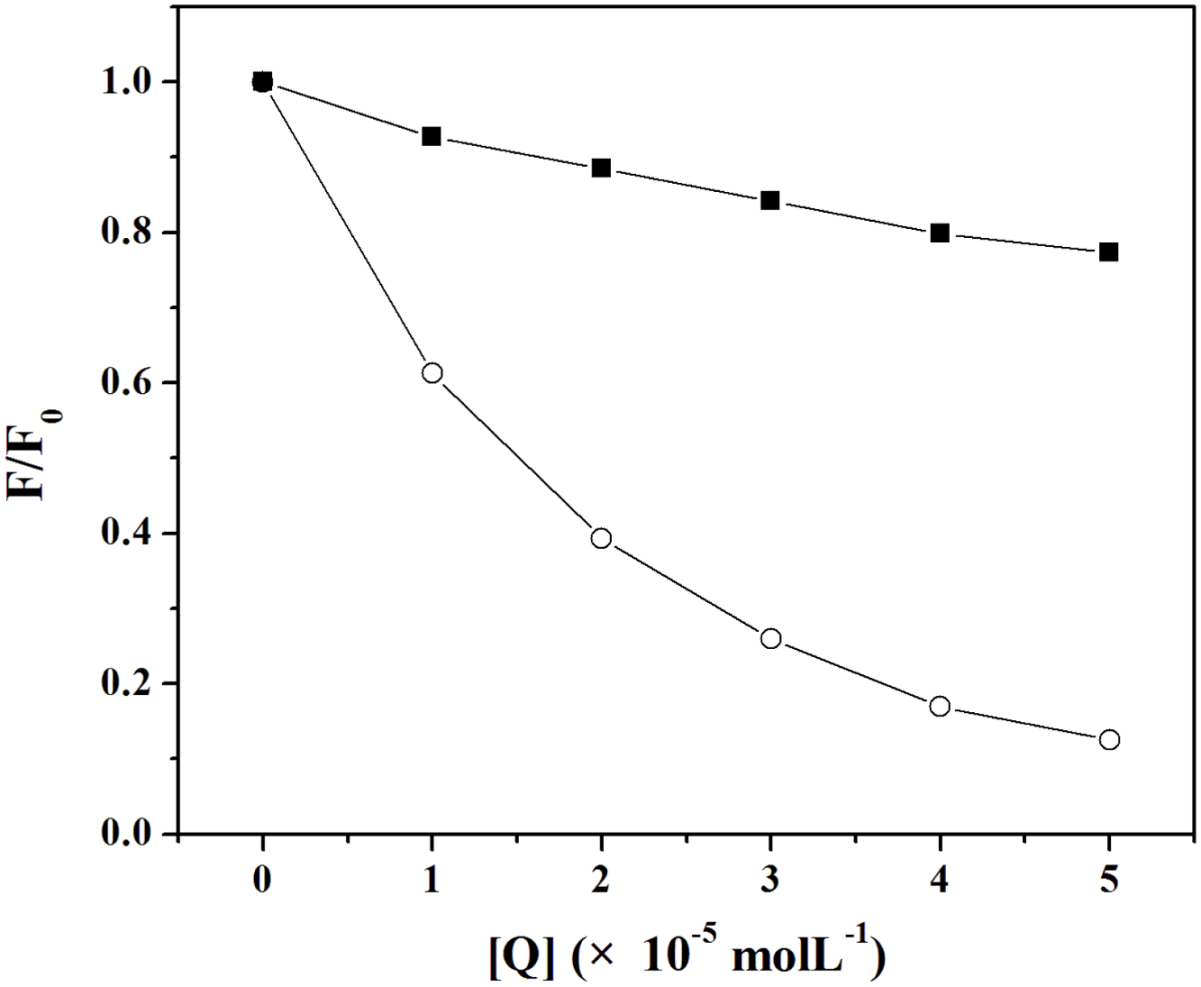
Quenching of Cu/ZnSOD synchronous fluorescence by MBI. Conditions: Cu/ZnSOD: 3.0×10^−6^ molL^−1^; (○)
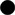
Δ*λ* = 15 nm and (▪) Δ*λ* = 60 nm.

With the increasing concentration of MBI, the emission maximum of tyrosine residues (in Figure 5A) and tryptophan residues (in Figure 5B) had blue shifts, demonstrating that the conformation of Cu/ZnSOD was changed such that the polarity around the tyrosine and tryptophan residues decreased and the hydrophobicity increased. It is shown in Figure 6 that the slope was higher when Δ*λ* was 15 nm revealing that MBI was closer to the tyrosine residues than to the tryptophan residues. Because the binding site of MBI was closer to the tyrosine residues, the microenvironments of the tyrosine residues were influenced more than those of the tryptophan residues.

#### Circular dichroism

**Figure 7:**
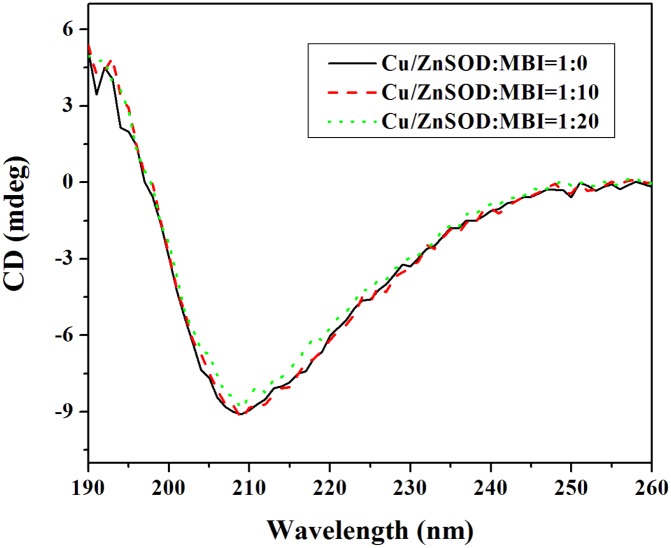
CD spectra of MBI-Cu/ZnSOD system. Conditions: Cu/ZnSOD: 5.0×10^−7^ molL^−1^; pH 7.4; *T* = 293 K.

To further ascertain the possible influence of MBI on the secondary structure of Cu/ZnSOD, CD spectroscopy was performed. The CD spectra of Cu/ZnSOD treated by various concentration of MBI were detected in Figure 7. We have performed the analysis of CD spectra by using the CDPro software package and summarized the results from the CD analysis for four secondary structures: α-helix, β-sheet, turns and unordered (Table 4) [42]. Cu/ZnSOD has the secondary structures of 8.3% α-helix, 39.0% β-sheet, 21.8% turns and 31.1% unordered. With the addition of MBI to Cu/ZnSOD (1∶20), the α-helix decreased by 0.5%, the β-sheet increased by 0.7%, turns increased 0.4% and unordered section decreased by 0.7%. The results indicated that the binding of MBI with Cu/ZnSOD induced some secondary structure changes in Cu/ZnSOD. On the basis of the above experimental results, the binding of MBI to Cu/ZnSOD induced conformational changes in Cu/ZnSOD and MBI had an obvious denaturing effect on Cu/ZnSOD.

**Table 4:** Effects of MBI on the percentage of secondary structural elements in Cu/ZnSOD at 293 K.

Molar ratio ofCu/ZnSOD to MBI	Secondary structural elements in Cu/ZnSOD			
	α-helix	β-sheet	turns	unordered
1∶0	8.3%	39.0%	21.8%	31.1%
1∶10	8.2%	38.9%	21.9%	31.0%
1∶20	7.8%	39.7%	22.2%	30.4%

### Effect of MBI on Cu/ZnSOD activity

**Figure 8:**
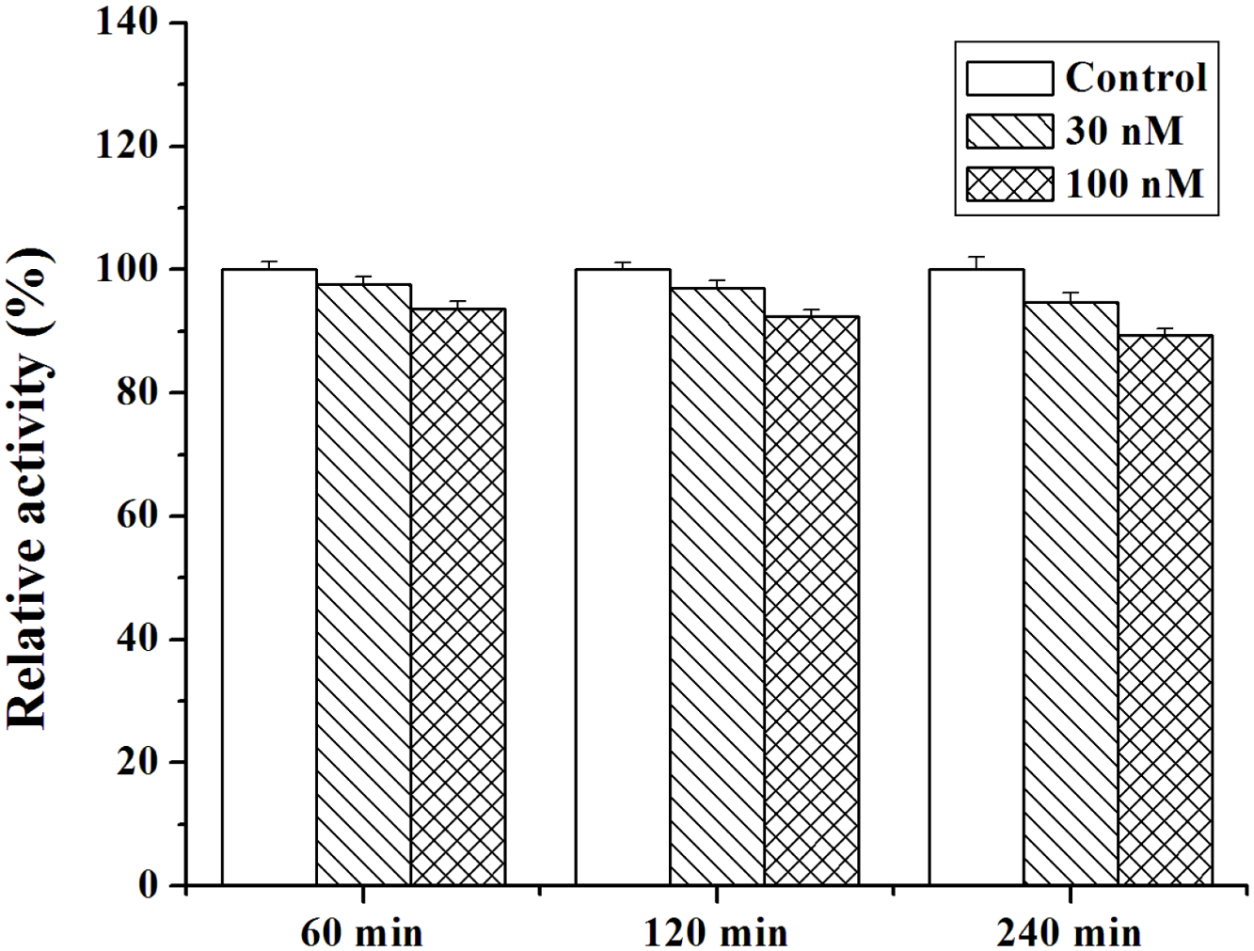
Effect of MBI on Cu/ZnSOD activity. Cu/ZnSOD was exposed to graded concentrations of MBI for 60, 120 or 240 min. Conditions: Cu/ZnSOD: 3 nM. Data were pooled from at least three independent experiments and analyzed with one-way ANOVA. Error bars indicate SD.

The structure of a protein is related to the function such that a structural variation may affect its normal physiological function. It can be seen from the above data that the effect of MBI on Cu/ZnSOD conformation is obvious. Therefore, the effects of different concentrations of MBI on the activity of Cu/ZnSOD in vitro at physiological pH 7.4 were investigated (Figure 8). The relative Cu/ZnSOD activities were reduced to 97.5±1.2 and 93.7±1.2% after treatment with 30 and 100 nM MBI for 60 min. As the exposure time increased to 240 min, the activities were reduced to 94.7±1.5 and 89.3±1.2%. The results indicated that the MBI concentration had a significant effect on Cu/ZnSOD activities. However, the incubation time had little effect on the enzyme activities. Based on the docking result, MBI did not directly bind into the Cu/ZnSOD activity site, but the binding of MBI into the enzyme interface of two subdomains influenced the microenvironments of the Cu/ZnSOD activity sites which resulted in the reduced Cu/ZnSOD activity.

## Conclusions

In this paper, the toxic interaction of MBI with Cu/ZnSOD was carried out by multiple spectroscopic and molecular docking methods under simulated physiological conditions. MBI can spontaneously bind with Cu/ZnSOD to form a MBI-Cu/ZnSOD complex with one binding site through hydrogen bonds and van der Waals forces. The UV-visible absorption, synchronous fluorescence and CD spectra indicated that the microenvironment and secondary structure of Cu/ZnSOD were altered in presence of MBI. MBI bound into the Cu/ZnSOD interface of two subdomains. Because the binding of MBI affected the microenvironment of the Cu/ZnSOD activity site, MBI led to the inhibition of Cu/ZnSOD activity. This work provides important insights into the interaction mechanism of MBI with Cu/ZnSOD.

## Supporting Information

Figure S1 Plot of lg[(*F*_0_–*F*)/*F*] versus lg[*Q*] for the interaction of MBI and Cu/ZnSOD at 293 and 310 K.
(DOC)

## References

[1] Reuter S, Gupta SC, Chaturvedi MM, Aggarwal BB (2010) Oxidative stress, inflammation, and cancer: how are they linked? Free Radical Bio Med 49: 1603–1616. 20840865 10.1016/j.freeradbiomed.2010.09.006 PMC2990475

[2] Kehrer JP (2000) The Haber-Weiss reaction and mechanisms of toxicity. Toxicology 149: 43–50. 10963860 10.1016/s0300-483x(00)00231-6

[3] Trpkovic A, Todorovic-Markovic B, Trajkovic V (2012) Toxicity of pristine versus functionalized fullerenes: mechanisms of cell damage and the role of oxidative stress. Arch Toxicol 86: 1809–1827. 22562437 10.1007/s00204-012-0859-6

[4] Zong WS, Liu RT, Sun F, Wang MJ, Zhang PJ, et al (2010) Cyclic voltammetry: A new strategy for the evaluation of oxidative damage to bovine insulin. Protein Sci 19: 263–268. 20027620 10.1002/pro.313 PMC2865718

[5] Kocer M, Naziroglu M, Kocer G, Sonmez TT (2014) Effects of bisphosphonate on oxidative stress levels in patients with different types of cancer. Cancer Invest 32: 37–42. 24308847 10.3109/07357907.2013.861475

[6] Subapriya R, Kumaraguruparan R, Ramachandran CR, Nagini S (2002) Oxidant-antioxidant status in patients with oral squamous cell carcinomas at different intraoral sites. Clin Biochem 35: 489–493. 12413611 10.1016/s0009-9120(02)00340-5

[7] Aaseth J, Stoa-Birketvedt G (2000) Glutathione in overweight patients with poorly controlled type 2 diabetes. J Trace Elem Exp Med 13: 105–111.

[8] Banaclocha MM, Hernandez AI, Martinez N, Ferrandiz ML (1997) N-acetylcysteine protects against age-related increase in oxidized proteins in mouse synaptic mitochondria. Brain Res 762: 256–258. 9262186 10.1016/s0006-8993(97)00493-9

[9] Strasser EM, Wessner B, Manhart N, Roth E (2005) The relationship between the anti-inflammatory effects of curcumin and cellular glutathione content in myelomonocytic cells. Biochem Pharmacol 70: 552–559. 16002051 10.1016/j.bcp.2005.05.030

[10] Shinomiya K, Fukunaga M, Kiyomoto H, Mizushige K, Tsuji T, et al (2002) A role of oxidative stress-generated eicosanoid in the progression of arteriosclerosis in type 2 diabetes mellitus model rats. Hypertens Res 25: 91–98. 11924732 10.1291/hypres.25.91

[11] Amer J, Ghoti H, Rachmilewitz E, Koren A, Levin C, et al (2006) Red blood cells, platelets and polymorphonuclear neutrophils of patients with sickle cell disease exhibit oxidative stress that can be ameliorated by antioxidants. Brit J Haematol 132: 108–113. 16371026 10.1111/j.1365-2141.2005.05834.x

[12] Giustarini D, Dalle-Donne I, Tsikas D, Rossi R (2009) Oxidative stress and human diseases: Origin, link, measurement, mechanisms, and biomarkers. Crit Rev Cl Lab Sci 46: 241–281. 10.3109/10408360903142326 19958214

[13] Sies H (1991) Oxidative stress: from basic research to clinical application. Am J Med 91: 31S–38S. 10.1016/0002-9343(91)90281-2 1928209

[14] Miao L, St Clair DK (2009) Regulation of superoxide dismutase genes: implications in disease. Free Radical Bio Med 47: 344–356. 19477268 10.1016/j.freeradbiomed.2009.05.018 PMC2731574

[15] Finsgar M (2013) 2-Mercaptobenzimidazole as a copper corrosion inhibitor: Part I. Long-term immersion, 3D-profilometry, and electrochemistry. Corrosion Sci 72: 82–89.

[16] Finsgar M (2013) 2-Mercaptobenzimidazole as a copper corrosion inhibitor: Part II. Surface analysis using X-ray photoelectron spectroscopy. Corrosion Sci 72: 90–98.

[17] Cheng XL, Li QB, Liang HB (1999) Analysis of organic additives in copper-plating brightener by high performance liquid chromatography. Chinese J Chromatogr 17: 602–603. 12552707

[18] Sakemi K, Ito R, Umemura T, Ohno Y, Tsuda M (2002) Comparative toxicokinetic/toxicodynamic study of rubber antioxidants, 2-mercaptobenzimidazole and its methyl substituted derivatives, by repeated oral administration in rats. Arch Toxicol 76: 682–691. 12451444 10.1007/s00204-002-0392-0

[19] Rastegarzadeh S, Rezaei ZB (2013) Environmental assessment of 2-mercaptobenzimidazole based on the surface plasmon resonance band of gold nanoparticles. Environ Monit Assess 185: 9037–9042. 23657736 10.1007/s10661-013-3233-0

[20] Khramov A, Voevodin NN, Balbyshev VN, Mantz RA (2005) Sol-gel-derived corrosion-protective coatings with controllable release of incorporated organic corrosion inhibitors. Thin Solid Films 483: 191–196.

[21] Jungclaus GA, Lopez-Avila V, Hites RA (1978) Organic compounds in an industrial Wastewater: a case study of their environmental impact. Environ Sci Technol 12: 88–96.

[22] Spies R, Andresen B, Brian D, David W, Lawrence J (1987) Benzthiazoles in estuarine sediments as indicators of street runoff. Nature 327: 697–699.

[23] Airaudo CB, Gayte-Sorbier A, Momburg R, Laurent P (1990) Leaching of antioxidants and vulcanization accelerators from rubber closures into drug preparations. J Biomat Sci Polym Ed 1: 231–241. 10.1163/156856289x00127 2279005

[24] Kawasaki Y, Umemura T, Saito M, Momma J, Matsushima Y, et al (1998) Toxicity study of a rubber antioxidant, 2-mercaptobenzimidazole, by repeated oral administration to rats. J Toxicol Sci 23: 53–68. 9513921 10.2131/jts.23.53

[25] Gaworski CL, Aranyi C, Vana S, Rajendran N, Abdo K, et al (1991) Prechronic inhalation toxicity studies of 2-mercaptobenzimidazole (2-Mbi) in F344/N rats. Fundam Appl Toxicol 16: 161–171. 2019341 10.1016/0272-0590(91)90144-s

[26] Doerge DR (1986) Mechanism-based inhibition of lactoperoxidase by thiocarbamide goitrogens. Biochemistry 25: 4724–4728. 3768307 10.1021/bi00364a041

[27] Yamano T, Noda T, Shimizu M, Morita S (1995) The adverse-effects of oral 2-mercaptobenzimidazole on pregnant rats and their fetuses. Fundam Appl Toxicol 25: 218–223. 7665005 10.1006/faat.1995.1057

[28] Morris GM, Huey R, Lindstrom W, Sanner MF, Belew RK, et al (2009) AutoDock4 and AutoDockTools4: Automated docking with selective receptor flexibility. J Comput Chem 30: 2785–2791. 19399780 10.1002/jcc.21256 PMC2760638

[29] Vilar S, Cozza G, Moro S (2008) Medicinal chemistry and the molecular operating environment (MOE): application of QSAR and molecular docking to drug discovery. Curr Top Med Chem 8: 1555–1572. 19075767 10.2174/156802608786786624

[30] Ma LL, Ze YG, Liu J, Liu HT, Liu C, et al (2009) Direct evidence for interaction between nano-anatase and superoxide dismutase from rat erythrocytes. Spectrochim Acta A 73: 330–335. 10.1016/j.saa.2009.02.041 19345606

[31] Beauchamp C, Fridovich I (1971) Superoxide dismutase: improved assays and an assay applicable to acrylamide gels. Anal Biochem 44: 276–287. 4943714 10.1016/0003-2697(71)90370-8

[32] Sun Y, Oberley LW, Li Y (1988) A simple method for clinical assay of superoxide dismutase. Clin Chem 34: 497–500. 3349599

[33] Zhou YL, Liao JM, Du F, Liang Y (2005) Thermodynamics of the interaction of xanthine oxidase with superoxide dismutase studied by isothermal titration calorimetry and fluorescence spectroscopy. Thermochimica Acta 426: 173–178.

[34] Zhang YZ, Zhou B, Zhang XP, Huang P, Li CH, et al (2009) Interaction of malachite green with bovine serum albumin: Determination of the binding mechanism and binding site by spectroscopic methods. J Hazard Mater 163: 1345–1352. 18786760 10.1016/j.jhazmat.2008.07.132

[35] Hossain M, Khan AY, Kumar GS (2011) Interaction of the anticancer plant alkaloid sanguinarine with bovine serum albumin. PLoS One 6: 12. 10.1371/journal.pone.0018333 PMC3071820 21494677

[36] Lakowicz JR, Weber G (1973) Quenching of protein fluorescence by oxygen. Detection of structural fluctuations in proteins on the nanosecond time scale. Biochemistry 12: 4171–4179. 4200894 10.1021/bi00745a021 PMC6945976

[37] Ware WR (1962) Oxygen quenching of fluorescence in solution: an experimental study of the diffusion process. J Phys Chem 66: 455–458.

[38] Gokara M, Sudhamalla B, Amooru DG, Subramanyam R (2010) Molecular interaction studies of trimethoxy flavone with human serum albumin. PLoS One 5: 9. 10.1371/journal.pone.0008834 PMC2809094 20098677

[39] Ross PD, Subramanian S (1981) Thermodynamics of protein association reactions: forces contributing to stability. Biochemistry 20: 3096–3102. 7248271 10.1021/bi00514a017

[40] Wu T, Wu Q, Guan S, Su H, Cai Z (2007) Binding of the environmental pollutant naphthol to bovine serum albumin. Biomacromolecules 8: 1899–1906. 17407349 10.1021/bm061189v

[41] Chi ZX, Liu RT, Yang HX, Shen HM, Wang J (2011) Binding of tetracycline and chlortetracycline to the enzyme trypsin: spectroscopic and molecular modeling investigations. PLoS One 6: 9. 10.1371/journal.pone.0028361 PMC3242759 22205948

[42] Sreerama N, Woody RW (2004) On the analysis of membrane protein circular dichroism spectra. Protein Sci 13: 100–112. 14691226 10.1110/ps.03258404 PMC2286510

