# Supplementary figures and images for: Molecular Interaction Mechanism between 2-Mercaptobenzimidazole and Copper-Zinc Superoxide Dismutase
Source: PLoS One. 2014 Aug 26;9(8):e106003. doi: 10.1371/journal.pone.0106003 (PMC4144957; doi:10.1371/journal.pone.0106003)

**Supporting Information:**


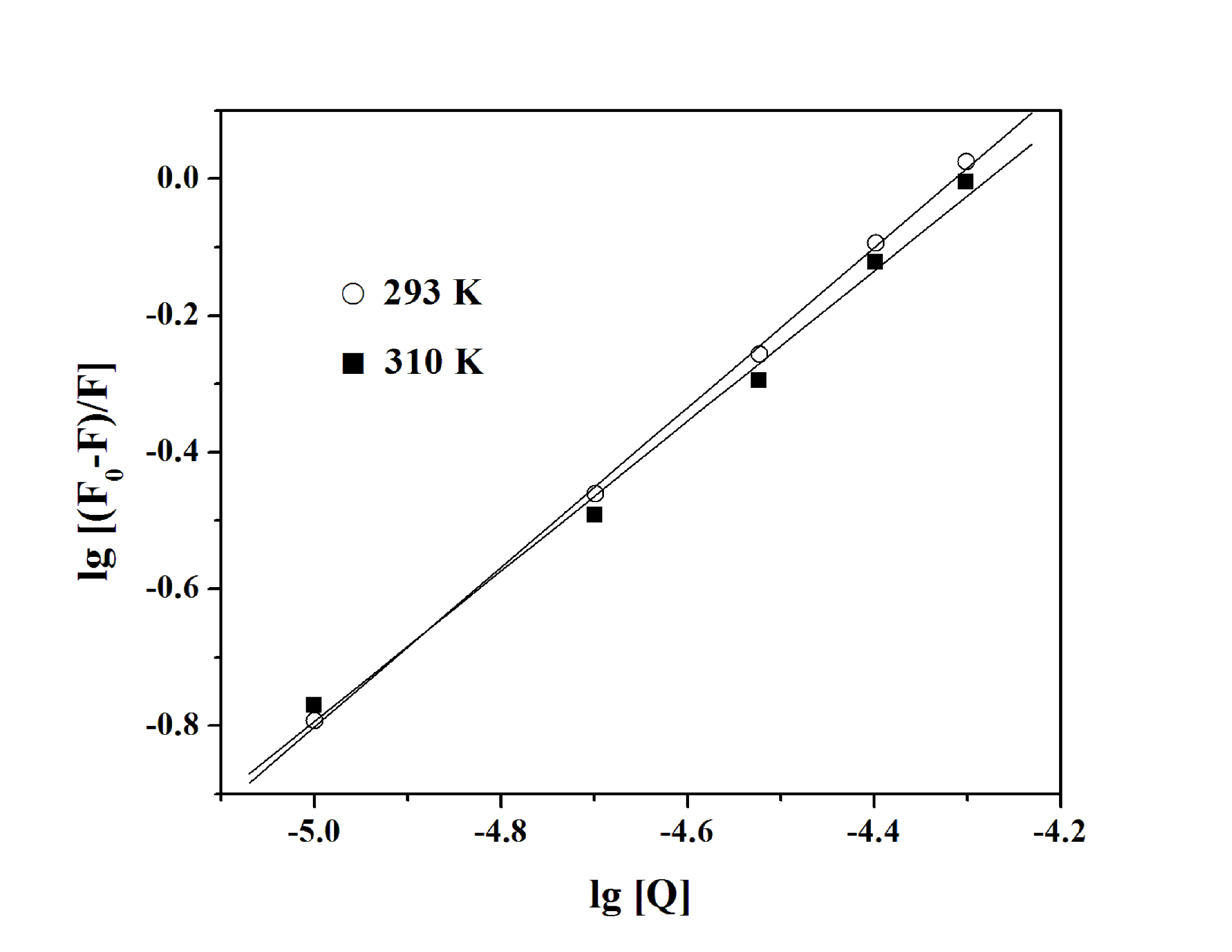


**Figure S1**

Supplement: Figure S1 — Plot of lg[(F 0–F)/F] versus lg[Q] for the interaction of MBI and Cu/ZnSOD at 293 and 310 K. (DOC) [file pone.0106003.s001.doc]
